# Coarsening behaviour of M_23_C_6_ carbides in creep-resistant steel exposed to high temperatures

**DOI:** 10.1038/srep29734

**Published:** 2016-07-13

**Authors:** M. Godec, D. A. Skobir Balantič

**Affiliations:** 1 Institute of Metals and Technology, Lepi pot 11, 1000 Ljubljana, Slovenia

## Abstract

High operating temperatures can have very deleterious effects on the long-term performance of high-Cr, creep-resistant steels used, for example, in the structural components of power plants. For the popular creep-resistant steel X20CrMoV12.1 we analysed the processes of carbide growth using a variety of analytical techniques: transmission electron microscopy (TEM) and diffraction (TED), scanning electron microscopy (SEM), and electron backscatter diffraction (EBSD). The evolution of the microstructure after different aging times was the basis for a much better understanding of the boundary-migration processes and the growth of the carbides. We present an explanation as to why some locations are preferential for this growth, and using EBSD we were able to define the proper orientational relationship between the carbides and the matrix.

The 9–12% Cr ferritic-martensitic steels are used in the steam-power-plant industry as structural materials (steel pipes, high-temperature boilers, etc.) because of their good corrosion and oxidation resistance as well as their excellent creep properties at high temperatures^1^. These high-Cr ferritic steels subjected to normalizing and tempering have a tempered lath martensitic microstructure consisting of elongated sub-grains (a high density of dislocations) and a dispersion of fine carbides (mostly M_23_C_6_) and carbonitrides (MX) along the sub-grain boundaries and within the matrix^2^,^3^,^4^. Recent investigations^5^,^6^ showed that the role of the M_23_C_6_ is more important than the role of the MX precipitates in the control of the sub-grain coarsening. These carbides are also the most stable form of carbides for a given chemical composition of the investigated steel, which means that their Gibbs free energy (ΔG_0_) is the lowest. The small M_23_C_6_ particles close to the boundaries are the major obstacles to the migration of the sub-grain boundaries and the gliding of the mobile dislocations, and therefore make a very important contribution to the long-term creep strength.

However, it is well known that a higher service temperature (600–650 °C) accelerates all the degradation processes in steels, such as the migration of atoms and dislocations, recovery and recrystallization, grain and sub-grain coarsening, as well as the coarsening of precipitates and martensite laths^7^. With an increasing exposure time the substitutional alloying elements, such as Cr, Mo, V and Mn, replace the Fe in the M_23_C_6_ carbides. Since this change of the chemical composition and the growth of the carbide particles are directly related to the diffusivity of the alloying elements and the self-diffusion of iron, which all depend greatly on the temperature, a change in the size of the particles and the particle-to-particle distance (L) is to be expected^8^,^9^. This leads to a larger average particle size and a reduction in the number of particles, both of which have a negative effect on the mechanical properties of the steel.

Here we present an SEM-, TEM- and EBSD-based investigation to explain the processes of martensite lath coarsening and the migration of the sub-grains at high temperature with respect to the growth of carbides in favourable positions.

## Experimental Details

### Material and specimen preparation

The investigated material is a 12% Cr tempered martensite steel X20CrMoV12.1 (German grade X20). The chemical composition is given in Table 1. The specimens were prepared from the wall of an industrial pipe (the diameter of which was 42 mm and the wall thickness, 4.5 mm) taken from service. The specimens were first normalized at 1040 °C and then quenched into oil according to the heat-treatment details prescribed by the steel producer. This temperature ensured the solution of all the carbide particles in the austenite. In order to simulate the evolution sequence of the carbides, the specimens were tempered at 800 °C for different times (3, 7, 24 and 168 h). This tempering temperature is well below the transformation temperature for α-γ (Ac_3_), which was determined to be 832 °C for this steel based on a dilatometry test. The microstructures examined by SEM were etched using iron (III) chloride.

A thin layer of carbon was evaporated onto the surface of the samples for the TEM investigations. Etching with a 10% solution of bromine-methanol was used to remove the deposited replica layer with embedded carbide particles.

### Analysis

The microstructures of the sample in the initial state (tempered at 730 °C for 30 minutes) and the samples tempered at 800 °C were investigated using a JEOL JSM 6500 F field-emission scanning electron microscope equipped with a HKL Nordlys II electron backscatter diffraction (EBSD) camera using Channel5 software. The instrument was operated at 15 kV and a 1.3-nA current for the EBSD analysis, with a tilting angle of 70 degrees. Individual diffraction patterns were obtained together with mapping of the areas of interest. Detection was set to 5–7 bands, with 4 × 4 binning. Minimal post-processing was performed in the case of the mappings, which was limited to removing the so-called “wild spikes”.

TEM micrographs and diffraction images were taken from extraction replicas using a JEOL AEM 2000 FX transmission electron microscope with an accelerating voltage of 200 kV.

Graphical processing software and 3.1 PRO image-analysis software were used to colour-code the areas occupied by the carbides. This was the basis for the determination of their volume fraction and other statistical microstructure parameters obtained for each annealed sample on 10 randomly chosen areas of 12 × 9 μm^2^.

Accelerated creep tests were performed at a temperature 580 °C with a stress of 170 MPa and for the testing time of 100 hours. These testing parameters were determined earlier to show the effect of the carbide particle distributions on the accelerated creep resistance.

## Results and Discussion

### Microstructure evolution during annealing

**Figure 1:**
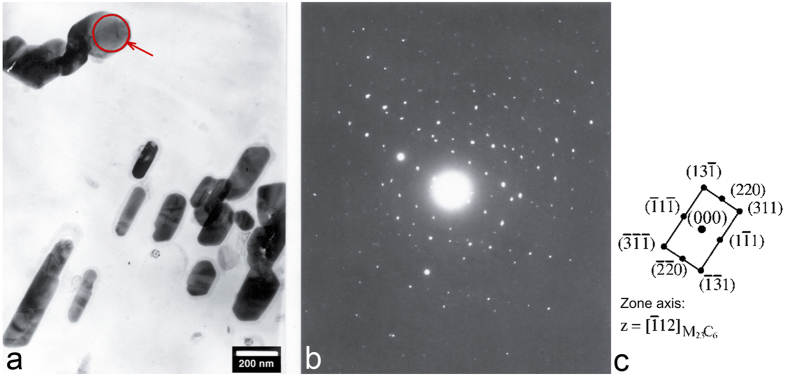
TEM analysis of precipitates in steel after annealing at 730 °C for 30 minutes; (**a**) extraction replica, (**b**) diffraction pattern and (**c**) indexed diffraction pattern for M_23_C_6_.

Steels with 9–12% Cr contain several precipitate types, such as M_23_C_6_, MX and Laves phases, which form either during the final normalizing and tempering heat treatment or during subsequent creep exposure^10^. Many of the formed precipitates are metastable and will disappear with time or are replaced by more stable ones. A well-known example of a typical precipitation sequence in 9–12% Cr steel is as follows^11^: matrix→ε-Fe_2.4_C→Fe_3_C + M_7_C_3_→M_23_C_6_→M_6_C. The formation of M_23_C_6_ was found to occur after just a short time, for example, after annealing for 30 minutes at 730 °C (Fig. 1), and remains in the microstructure even after 168 hours of annealing at 800 °C. The M_23_C_6_ was detected by transmission electron diffraction (TED), and Fig. 1b,c shows the zone axis [−112] of a cubic structure. The a_0_ for this cubic structure was determined from d-spacing measurements in diffraction image to be 1.034 nm. This corresponds to cubic M_23_C_6_^12^, where the M consists predominantly of chromium with a significant proportion of iron and several other carbide-forming elements in lower concentrations (Table 2).

**Figure 2:**
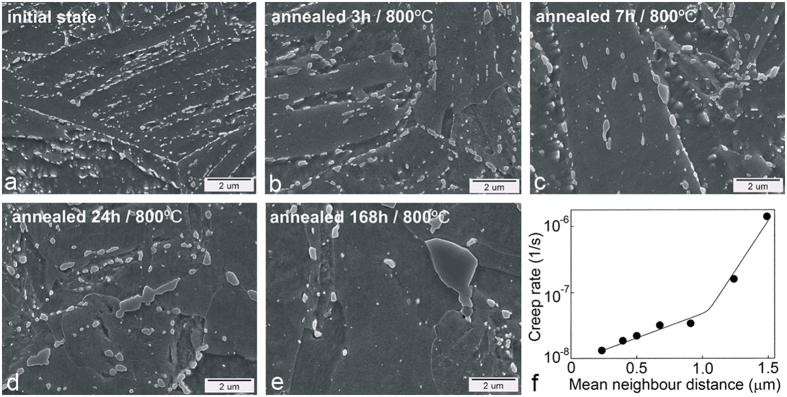
The SE images of microstructure evolution for different times and temperatures of annealing and corresponding results of creep test; (**a**) initial state quenched at 1040 °C and tempered at 730 °C for 30 minutes, (**b**) annealed 3 h/800 °C, (**c**) annealed 7 h/800 °C, (**d**) annealed 24 h/800 °C, (**e**) annealed 168 h/800 °C, (**f**) mean neighbour distance vs creep rate.

The working temperature (up to 650 °C) and the exposure under stress during service promote microstructural changes in the sub-grains and precipitates due to diffusion processes. In order to simulate the working conditions the specimens were exposed to a higher temperature to accelerate the precipitation processes, the growth of the carbides and the migration of the sub-grains. Figure 2 shows the microstructure evolution from the initial state to different exposure times at elevated temperature.

The microstructure of the quenched-only specimen (analysed by SEM) has a lath martensitic microstructure with almost no visible carbides. The initial state shows a typical quenched-and-tempered microstructure with fine-grained carbides distributed along prior-austenite grains and along the martensite laths (Fig. 2a). This was sufficient for the tetragonal crystal lattice of martensite to transform to a ferrite crystal structure, but the martensite morphology remains. Even a short annealing time (3 h/800 °C) causes the carbides to grow preferentially on the grain boundaries and the martensite lath boundaries (Fig. 2b). Besides the carbides’ growth, the migration of the sub-grains also occurs, which then leads to martensite lath coarsening. The carbides inside the coarse martensite laths disappear, while the carbides along the grain boundaries keep growing (Fig. 2c). The carbides’ growth on the grain boundaries is probably kinetically favoured, either because the elements necessary for their formation are more readily available in the matrix or their nucleation is easier, compared to those in the martensite laths, because the grain-boundary diffusion is much faster compared to the bulk diffusion^13^. The carbide-forming elements have to diffuse to the carbides, so their growth is dependent on the diffusion rate. Longer annealing times cause further coarsening of the carbides (Fig. 2d,e). It is clear that the number of carbides decreases while the volume fraction remains almost the same (Fig. 2a–e). Carbides located close to each other at favourable positions for growth can come into contact with each other during annealing and grow further as a single, larger carbide (Fig. 2d). A longer annealing time (Fig. 2e) causes a further migration of the grain boundaries, and the carbides form mostly along the grain boundaries. The number of small carbides decreases during aging, although even after 168 hours at 800 °C a small number of them are still present in the microstructure. It is not obvious whether these small carbides are those for which the size is decreasing due to their unfavourable positions or they are newly formed during the aging. Larger carbides are usually found at the triple grain boundaries. As we found in previous research^14^,^15^ the carbides grow with a prolonged annealing time, their neighbouring distances increase, which leads to a reduction in all the mechanical properties and especially the creep strength (Fig. 2f). The critical neighbour distance under these experimental conditions was found to be approximately 1 μm, and at this point the creep rate changes significantly. This could point to an indirect method for assessing the state of critical components in power plants based on simple light microscopy replicas. Even though 1 μm is difficult to resolve with a light microscope, based on our results it is possible to correlate the mean neighbour distance to the maximum size of the carbide particles. At a critical mean neighbour distance of 1 μm the larger carbide areas in the range 2–3 μm can be observed using light microscopy.

Secondary-electron images were analysed using graphical software so that the carbides are white coloured and the rest of the microstructure is black. Such binary images were processed in order to obtain some statistical parameters for the carbides (number, perimeter, volume, etc.) that are shown in Table 3. Based on these results we can conclude that the volume fraction of the M_23_C_6_ carbides remains almost the same, although their number decreases with the annealing time. The slight increase in the volume fraction probably relates to the measurement uncertainty in the carbides’ volume fraction, tending to produce larger values for the smaller carbides. The mean neighbouring distance as well as the mean perimeter increase with the annealing time, which causes fewer obstacles to dislocation movements.

### Coarsening processes

**Figure 3:**
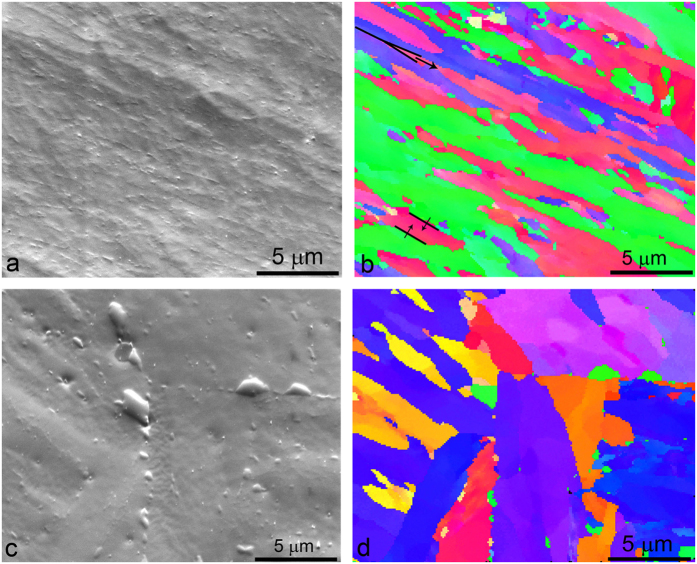
EBSD micrographs of the annealed specimens; (**a**) secondary electron image and, (**b**) IPF colouring in the Z direction of specimen annealed for 3 h at 800 °C, (**c**) secondary electron image and, (**d**) IPF colouring in the Z direction of specimen annealed for 168 h at 800 °C.

**Figure 4:**
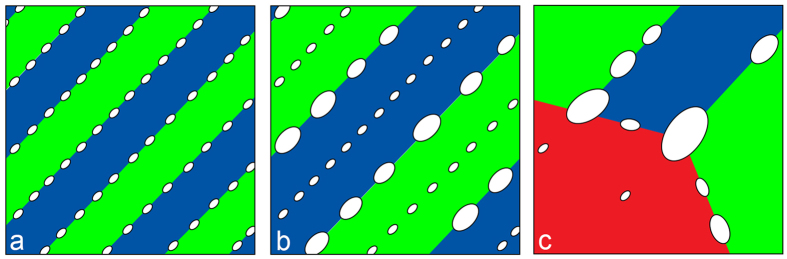
Scheme of the carbide-coarsening processes.

The SEM/EBSD analysis technique was used to gain information about the microstructure changes during annealing at 800 °C, as well as to learn more about the type of carbides and to determine the orientational relationship between the carbides and the matrix. Figure 3 clearly reveals the martensite lath growth, which from the lath structure transforms to the ferrite grains, some of them having a polygonal appearance and some of them still having a lath appearance. The EBSD IPF image indicates the grain-boundary migration (in Fig. 3b marked by arrows) that occurs in two ways: at the Y junctions it follows the zipper principle, and at the parallel boundaries, there is recombination of the two boundaries. After 168 hours of annealing at 800 °C the shape of the grains becomes more polygonal. At this point it is interesting to note that the majority of the carbides have the same orientation (Fig. 3d). However, for basic energetic reasons the carbides do not change their orientation during growth. Therefore, it can be concluded that their orientation remains the same as at the very beginning. Inside the cluster of martensite laths the carbides precipitate with the same orientation, which correlates with the orientation of the martensite lath. There are no large carbides on the simple grain boundaries; there are only carbides on the triple grain boundaries. The M_23_C_6_ carbides are rich in chromium; therefore, the chromium has to diffuse to the carbide and the carbide growth is defined by the chromium diffusion. The diffusion of chromium along the grain boundaries is much faster and therefore the carbides grow much more quickly, particularly at the triple-junction borders. A schematic presentation of the carbides’ coarsening and the grain-boundary migration is shown in Fig. 4.

### Orientational relationship

**Figure 5:**
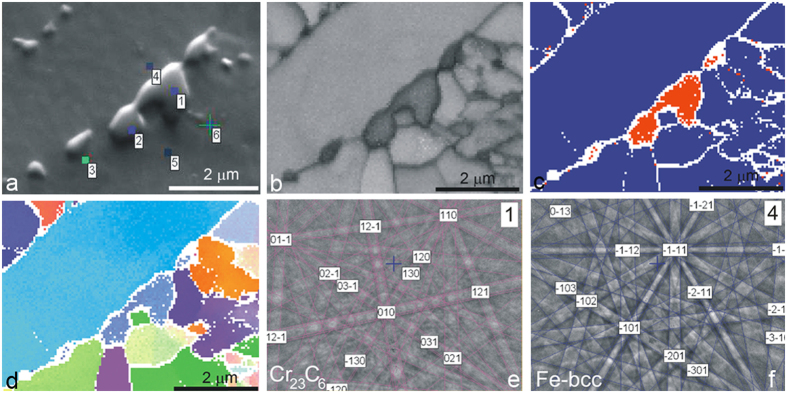
EBSD mapping of carbides after 168 hours of annealing at 800 °C; (**a**) SE image, (**b**) band-contrast image, (**c**) phase-map image, (**d**) IPF Z direction, (**e**) EBSD Kikuchi patterns of Fe ferrite, (**f**) EBSD Kikuchi patterns of Cr_23_C_6_.

In our case the EBSD analysis was made on carbides larger than 200 nm, even though a 15-kV accelerating voltage was used. The reason for this is the topography, due to the hardness differences between the carbides and the matrix. A typical area of a carbides cluster along a grain boundary of the specimen annealed 168 hours at 800 °C was chosen for the analysis shown in Fig. 5a. During the carbides’ growth some of them come into contact and grow as a series of three or more carbides. Figure 5b shows a band-contrast image where the carbides are seen as a darker phase. Figure 5c is an EBSD phase map image showing the ferrite phase as blue and the carbide phase as red. Figure 5d is IPF images in the Z directions showing the orientation of the phases. Based on the information from the EBSD Kikuchi patterns we found that the carbides are Cr_23_C_6_ (ref EBSD match factor) and the matrix is bcc Fe ferrite (Fig. 5e,f). The EBSD maps show that all the carbides in a cluster have the same orientation, as already explained by Fig. 3b.

**Figure 6:**
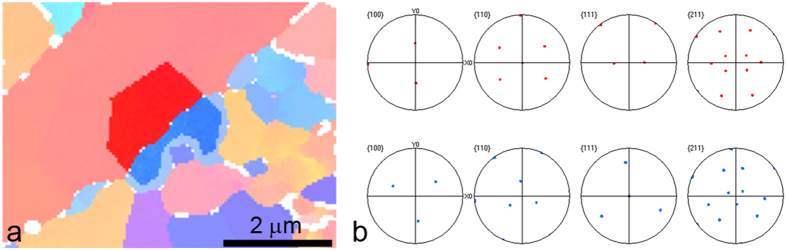
(**a**) Marked area of IPF image, (**b**) corresponding pole figures.

In the IPF image (Fig. 6a) the area of the carbides and the area of the neighbouring larger ferrite grain were marked and the corresponding pole figures were determined. After specimen annealing for 168 hours at 800 °C the carbides are in an orientational relationship with the neighbouring ferrite. The orientation relation is: {110}Fe-bcc//{111}Cr_23_C_6_, [110]Fe-bcc//[211]Cr_23_C_6_. Most probably the Cr_23_C_6_ precipitates in relation to the bcc-Fe matrix or the martensite matrix in such a way that certain planes are parallel and form the smallest mismatch. It is expected that the densest planes and the densest directions of the matrix and the growing carbides are in parallel, which is the situation in the Kurdjumov-Sachs orientation relationship^16^.

## Conclusions

Based on the TEM, SEM and EBSD analyses of the microstructure development during prolonged aging at 800 °C we proposed a model by which we can explain the carbide-coarsening processes in the steel. During aging the carbides grow, while the volume fraction remains almost the same, and the number of carbides decreases significantly. Consequently, the mean neighbouring distance increases, leading to a reduced creep resistance that can be identified with light microscopy. After a certain time at temperature the martensite laths start to coarsen, and as a consequence of the grain-boundary migration this occurs in two ways: first, by a recombination of two boundaries and, second, based on the zipper principle. For such a grain-boundary migration process some carbide stringers are caught in between the martensite laths. The diffusion of carbide-forming elements to the carbides that are not along grain boundaries is significantly slower. Therefore, their growth is negligible and over time they disappeared. Further grain-boundary migration favours the growth of carbides at the triple grain boundaries. The carbides formed at the grain boundaries are in a certain orientational relationship with the matrix and during aging this relationship does not change. The investigated Cr_23_C_6_ carbides are related to the bcc-Fe matrix or the martensite matrix in such a way that certain planes are parallel and form with the smallest mismatch.

## Additional Information

**How to cite this article**: Godec, M. and Skobir Balantič, D. A. Coarsening behaviour of M_23_C_6_ carbides in creep-resistant steel exposed to high temperatures. *Sci. Rep.* **6**, 29734; doi: 10.1038/srep29734 (2016).

## Figures and Tables

**Table 1:** Chemical composition of the investigated steel in wt.%.

Steel	C	Si	Mn	P	S	Cr	Mo	Ni	V
X20CrMoV12.1	0.18	0.24	0.51	0.009	0.014	11.7	0.96	0.66	0.27

**Table 2:** Chemical composition of the carbide analysed in Fig. 1 measured by EDS in at.%.

Element	at.%
Cr	64.40
Fe	22.99
Mn	7.44
Mo	2.63
V	2.54

**Table 3:** Statistical parameters of the carbides from Fig. 1.

	Area fraction of carbide phase [%]	Number of carbides per 100 μm^2^	Mean neighbour distance [μm]	Mean perimeter [μm]
Initial state	5.6	1231	0.06	0.16
Annealed 3 h/800 °C	5.8	408	0.24	0.80
Annealed 7 h/800 °C	5.9	280	0.29	0.85
Annealed 24 h/800 °C	6.8	197	0.46	2.03
Annealed 168 h/800 °C	6.6	116	0.68	2.61
